# Expression of AMPK and PLIN2 in the regulation of lipid metabolism and oxidative stress in bitches with open cervix pyometra

**DOI:** 10.1186/s12917-025-04622-1

**Published:** 2025-03-13

**Authors:** Xin Deng, Hui Liu, Wei Zhao, Rui Wu, Kuo Chen, Qing Li, Murat Onur Yazlık, Hüseyin Özkan, Jingyuan Ren, Jiacheng Zhang, Shiyi Liu, Ling Mei, Shangfeng Li, Jiasui Zhan, Binhong Hu

**Affiliations:** 1 College of Chemistry and Life Sciences, Chengdu Normal University, Chengdu, Sichuan 611130 PR China; 2 Department of Pathophysiology, School of Basic Medical Sciences, College of Medicine, Zhengzhou University, Zhengzhou, 450001 PR China; 3 The First Affiliated Hospital of Zhengzhou University, Zhengzhou, 450052 PR China; 4 Department of Obstetrics and Gynecology, Ankara University Faculty of Veterinary Medicine, Ankara, 06070 Turkey; 5 Faculty of Veterinary Medicine, Department of Genetics, Hatay Mustafa Kemal University, Hatay, 31060 Turkey; 6 Department of Forest Mycology and Plant Pathology, Swedish University of Agricultural Sciences, Uppsala, SE-75007 Sweden; 7 Zhi Pet Animal Hospital, Chengdu, Sichuan 611830 PR China

**Keywords:** AMPK, Lipid metabolism, Open cervix pyometra, Oxidative stress, PLIN2

## Abstract

The pathogenesis of canine pyometra is multifactorial, involving hormonal imbalances, aberrant immune responses, and metabolic dysregulation includes lipid metabolism and oxidative stress. This study focuses on lipid metabolism and oxidative stress, revealing the key regulatory role of AMPK and PLIN2 in canine pyometra. Bitches with open cervix pyometra (n:8) and healthy bitches undergoing elective ovariohysterectomy (n:4) were enrolled to the study. In experiment one, the serum and tissue levels of Malondialdehyde (MDA) and Superoxide Dismutase (SOD) activity were assessed. Additionally, uterine histopathological analysis, AMPK and PLIN2 expressions were determined through immunohistochemistry. Furthermore, inflammation, oxidative stress, and lipid metabolism-related factors were evaluated using Western blot analysis. In experiment two, primary cell cultures were prepared from healthy uterine endometrial cells of the dogs in control group. Cultured canine endometrial epithelial cells were treated with lipopolysaccharide (LPS) along with oleic acid (OA) to induce an inflammatory response. Tissue and serum MDA and SOD levels were greater in dogs with pyometra. Accumulated lipid droplets were observed in the uterine tissue of bitches with pyometra. The phosphorylation of AMPK and the expression of PLIN2 significantly increased in the pyometra group. The expression of related lipid synthesis proteins such as ACC1, FASN, SREBP-1c, and PLIN2 was upregulated, while PPARα and PGC1α were downregulated in bitches with pyometra. In experiment two, activation of AMPK and PLIN2 not only restores the expression of PGC1α, but also effectively alleviates inflammation and oxidative stress. The role of lipid metabolism and oxidative stress in canine pyometra is elucidated, thus contributing to the pathogenesis of pyometra in dogs.

**Supplementary Information:**

The online version contains supplementary material available at 10.1186/s12917-025-04622-1.

## Introduction

Pyometra is a disease of the uterus, even the etiology is not clearly understood the different degree degenerative changes under influence of physiological hormonal fluctuations during the estrous cycle enables to the bacteria mainly *Escherichia coli (E.coli)* to invade and grow in uterine tissue that leading to purulent exudate accumulation into the uterine lumen [1–3]. The *E.coli* has ability to produce endotoxin that trigger an immune response, recruit immune cells, and produce excessive inflammatory mediators as well as reactive oxygen species, thus disrupting the uterine microenvironment homeostasis [1, 4, 5]. The disruption of oxidant / antioxidant balance due to excessive reactive oxygen species (ROS) production leads to oxidative stress that is closely related to pyometra. Although the uterine tissue antioxidant enzyme superoxide dismutase activity decreases for the neutralization of ROS production [6]. Due to the damage of oxidative stress to the lipid moiety specifically polyunsaturated fatty acids, resulted with malondialdehyde (MDA) production which indicates considerable cellular damage in uterine tissues [7–9]. Thus, determining the role of lipid metabolism in canine pyometra may contribute to pathogenesis depending on the relationship between lipid metabolism products and oxidative stress.

When exposed to inflammatory stimuli, the lipid composition of the endometrium undergoes changes. Previous studies have reported abnormal cell proliferation, differentiation, and excessive accumulation of lipid droplets (LDs) in the infected endometrium due to Canine alterations in energy metabolism patterns [10]. Bacterial lipopolysaccharide could facilitate LDs formation in mammalian cells and increase the level of perilipin2 (PLIN2) and concentrate various host defense proteins and antimicrobial peptides on lipid droplets [11]. PLIN2 is associated with intracellular lipid metabolism and is expressed in the uterus. The high expression of PLIN2 not only promotes the formation and stability of LDs but also may alleviate oxidative stress damage by enhancing the antioxidant capacity of cells [12].

As a cellular energy regulator, AMP-activated Protein Kinase (AMPK) assumes a crucial role in the physiological and pathological processes of the endometrium [13–15]. Under energy stress, AMPK is activated to sustain metabolic equilibrium [13]. Its activation also gives rise to vasodilation and is regarded as a potential therapeutic approach for restoring uterine perfusion in pregnancy-related disorders [16]. Furthermore, as a custodian of metabolism and mitochondrial homeostasis, AMPK also possesses the functionality of regulating oxidative stress and lipid metabolism [17, 18]. Therefore, in canine pyometra, the AMPK/PLIN2 signaling might act as a significant regulatory role for modulating lipid storage and mobilization, optimizing cellular energy utilization, and influencing the regulatory mechanisms of oxidative stress and inflammatory responses.

The aim of this study was to determine the lipid metabolism, immune and oxidative stress markers in spontaneously developed canine pyometra tissue samples. Secondly, herein it was evaluated the possible role of AMPK and PLIN2 on immune and oxidative stress markers in canine endometrial cell culture which are treated by Lipopolysaccharide (LPS) with oleic acid (OA). Thus, in addition to contributing to the pathogenesis of pyometra, a new perspective and strategy can be provided for the prevention and treatment of pyometra.

## Materials and methods

### Experiment 1

The animal study was approved by the Animal Administration and Ethics Committee of Chengdu Normal University and Zhi Pet Animal Hospital (Approval number: CDNU of Canine Pyometra: 03-2023-017 C and 20230612ZP). The collection of canine uterine samples is conducted with the written consent of the dog owners, and all experiments adhere strictly to the Animal Ethical Procedures and Guidelines of the People’s Republic of China to ensure humane treatment throughout the process. The intravenous propofol dose was administered at 5 mg/kg, complemented by isoflurane inhalation anesthesia to maintain optimal animal welfare throughout the experiment. All procedures in this study were conducted in strict adherence to the ARRIVE guidelines (https://arriveguidelines.org).

#### Samples collection

A total of 12 intact female dogs were enrolled to the study between August 2023 and January 2024. Among them, four healthy bitches presented for elective ovariohysterectomy allocated to control group. The remaining eight female dogs were diagnosed with open cervix pyometra based on anamnesis (lethargy, depression, vaginal discharge), and clinical findings (blood tests) and abdominal ultrasonography (fluid filled uterus with variable wall thickness and proliferative changes). Serum samples for the determination of antioxidant enzyme activity were collected at the same time and centrifuged at 1300 × g at 4 °C for 10 min. The serum was transferred into 2 mL tubes and frozen − 80 °C until analysis.

Routine ovariohysterectomy and pyometra operations were performed under general anesthesia as previously described [19]. Preoperative medication for analgesia and preparation for the operation were performed as previously described [8]. Propofol was used for the induction of anesthesia, which was maintained with isoflurane delivered in oxygen [19]. Additionally, intravenous fluid therapy was administered as outlined in prior studies [9]. This standardized protocol ensured consistent and effective management of anesthesia and perioperative care. Uterine tissue samples were collected from the cranial, medial and caudal portions of the uterine horns for the determination of tissue antioxidant enzyme activity, histopathology and immunohistochemistry, western blot analysis under sterile conditions. Then the collected tissue samples were washed with Phosphate Buffered Saline (PBS) then were rapidly frozen in liquid nitrogen for future use. The remaining uterine tissues after samples were fixed in formalin at room temperature for 24 h before undergoing histological and immunohistochemical examinations.

#### Hematoxylin and Eosin (HE) staining

A part of uterine samples was fixed in 10% formalin for over 24 h and embedded in paraffin. Then, paraffin Sect. (5 μm thick) were stained with hematoxylin eosin (HE), and followed by histopathological observation under digital scanning of sections.

#### Oil red O (ORO) staining

The cryosections of uterine samples collected from healthy bitches and dogs with pyometra were subjected to Oil Red O (ORO) staining for general lipid visualization. According to the methods described in the literature [10]: the frozen sections were fixed with 4% neutral buffered formaldehyde for 10 min, followed by three washes with phosphate-buffered saline (PBS) for 5 min each. Subsequently, the samples were treated with 60% isopropanol for 2 min to remove excess water and stained with an ORO working solution for a duration of 10 min. Afterward, the samples underwent brief rinsing in 60% isopropanol 5 times, each time lasting for 2 s. They were then immersed in distilled water for a period of 3 min to eliminate non-specific staining. To counterstain the samples, they were exposed to hematoxylin solution for a duration of 3 min before being washed in tap water and finally sealed and covered using glycerol gelatin mounting medium. Under an optical microscope, red areas indicate the presence of neutral lipids. Image J and IHC Profiler plug-in were used for the counting of positives [10, 20].

#### Immunohistochemical staining

The paraffin sections were successively placed in three eco-friendly dewaxing solutions for 10 min, treated with ethanol for 5 min each, and finally washed with distilled water. Then, antigen retrieval was performed, the sections were washed with PBS three times for 5 min each. After that, the sections were incubated in 3% hydrogen peroxide in the dark for 25 min after PBS washing. 3% Bovine Serum Albumin (BSA) was used for 30 min of serum blocking. Then, the sections were incubated at 4 °C overnight with the first antibody: p-AMPK (Servicebio, China, 1:200), PLIN2 (Servicebio, China, 1:100). After washing, the HRP-labeled goat anti-rabbit IgG (Servicebio, China, 1:200) was added and incubated at room temperature for 50 min. 3,3’-Diaminobenzidine (DAB) was used for coloration, and the sections were rinsed with tap water to stop the coloration reaction. Then, the cell nuclei were counterstained with hematoxylin. Finally, the samples were dehydrated with alcohol and xylene and sealed for observation under white light microscopy (Leica DM 4000). Immunohistochemical staining scores were assessed semi-quantitively [21, 22]. Positivity and scoring of staining were performed based on the percentage values of the 10 fields at high magnification (X 400) and were categorized in four grading categories. Cells with < 10% staining were scored as negative staining (−, 1); cells with 10–49% staining were scored as (+, 2); cells with 50–74% staining were scored as (++, 3); and cells with 75–100% staining were scored as (+++, 4). The immunohistochemical staining scores of the cellular immune reactions were assessed by two independent assessors and the mean score of these assessors was calculated.

#### SOD and MDA detection

The frozen uterine tissue samples (60 mg) were thawed on ice and sliced then placed in a glass homogenizer. Use a pipette to add 1 mL of normal saline, fully grind on ice to make 10% tissue homogenate, and centrifuge at 4 ℃ at 4,500 rpm for 15 min. The supernatant was collected for analysis. At the same time frozen serum samples were thawed on ice and analyzed for determination of - superoxide dismutase activity (SOD) (Amylet Scientific, China) and malondialdehyde level (MDA) (Najing Wanmuchun Biotechnology Co., Ltd, China) by using commercially available ELISA kits. Each experimental group was subjected to four independent replicates and absorbance was measured at 450 nm.

#### Western blotting

Western blotting techniques were performed to detect the expression levels of AMPKα, p-AMPKα, TNF-α, IL-6, HO-1, NQO1, ACC1, FASN, SREBP-1c, PLIN2, PPARα, PGC1α and β-actin (CST, USA, 1:1000) in canine uterine tissue samples in both control and pyometra groups. A total of 60 mg of uterine tissue was homogenized in RIPA Lysis Buffer (Beyotime, China) containing phosphatases/protease inhibitors (Beyotime, China). The protein content was determined using a BCA kit (Servicebio, China) and equal amounts of protein samples were separated by electrophoresis. Protein transfer was conducted at a constant voltage of 80 V for 30 min followed by a constant voltage of 120 V for 40 min to migrate the proteins onto a PVDF membrane. After incubating with 5% bovine serum albumin at room temperature for 2 h, primary antibody reaction took place overnight in a refrigerator shaker at 4℃. Subsequently, the PVDF membrane was washed 4 times with TBST buffer and incubated at room temperature for 1.5 h for secondary antibody reaction. Finally, after 4 TBST washes, the electrochemiluminescence (ECL) development method along with Image J software were utilized to calculate the relative expression quantity to analyze the obtained results. Each experimental group was subjected to three independent replicates.

### Experiment 2

For subsequent cell experiments, the primary cell culture was prepared rapidly with part of healthy dog uterine tissue samples under sterile conditions.

#### Cell culture and treatments with LPS and OA

The uterine tissue from a healthy dog within 2 months of the last heat, rinse it thoroughly with a glass dish containing antibiotic saline solution. Surgical scissors are used to open the uterine horns and separate the endometrium from the myometrium, placing it into a small glass vial. Tissue pieces were cut approximately 1 mm, rinse thoroughly with antibiotic saline, and transfer to a 15 ml centrifuge tube. The samples were centrifugated at 1000 rpm for 5 min. Following centrifugation, the samples were subsequently washed with PBS twice or thrice. Subsequently, 3 to 4 times the tissue volume of 0.25% trypsin was added, and the mixture was incubated in a 37 °C water bath for 10 min. Then the samples were centrifugated again at 1000 rpm for 5 min and the supernatant discarded. A small amount of culture medium was added and the well mixed. Evenly the mixed tissue pieces were distributed in a cell culture flask at a density of approximately 5 pieces/cm².

The isolated canine endometrial epithelial cells were cultured in Dulbecco’s modified Eagle’s medium (DMEM, complete medium) (Gibco, C11885500BT, USA) supplemented with 10% fetal bovine serum (FBS) and 1% penicillin-streptomycin (PS) at a temperature of 37 °C and under a CO_2_ concentration of 5%. Culture medium was replaced every 2 days. On day 4, when the cell confluence reached approximately 70–80%, they were subjected to treatment with lipopolysaccharide (LPS) at concentrations ranging from 1 to 10 µg/mL, along with oleic acid (OA) at a concentration of 0.2 mM, for a duration of 24 h to induce an inflammatory response. The activator 5-Aminoimidazole-4-carboxamide ribonucleoside (AICAR) was used to activate AMPK, incubated at 1 mM concentration for 24 h [23], The plasmid for PLIN2 overexpression was purchased from Sangong Biotechnology (Shanghai) Co., Ltd. Subsequently, PLIN2 was transfected into canine endometrial epithelial cells according to the manufacturer’s instructions for the Lipofectamine™ 2000 transfection reagent (Thermofisher Scientific, CA) [24]. Each experimental group was subjected to three independent replicates.

#### Cellular lipid droplet assay

After culturing and treating the cells on a slide, they were fixed with 4% paraformaldehyde for 10 min. Cellular experiments were performed according to the instructions of the Lipid Droplets Green Fluorescence Detection Kit (BODIPY 493/503) (Beyotime, China). BODIPY 493/503 and Hoechst 33,342 were used for live cell staining. Finally, the resulting images were observed and recorded under a fluorescence microscope.

### Statistical analysis

The experimental data were independently repeated at least three times and presented as mean ± standard deviation and analyzed using GraphPad Prism 9.5.0 statistical software. All data were initially tested for normal distribution using the Shapiro - Wilk test. Homogeneity of variances was verified by F test and Bartlett’s test. The *T*-test and one-way analysis of variance (ANOVA) was employed for group comparisons, with statistical significance defined as *p* < 0.05.

## Results

### Experiment 1

#### Superoxide dismutase activity and malondialdehyde levels altered under influence of canine pyometra

**Fig. 1:**
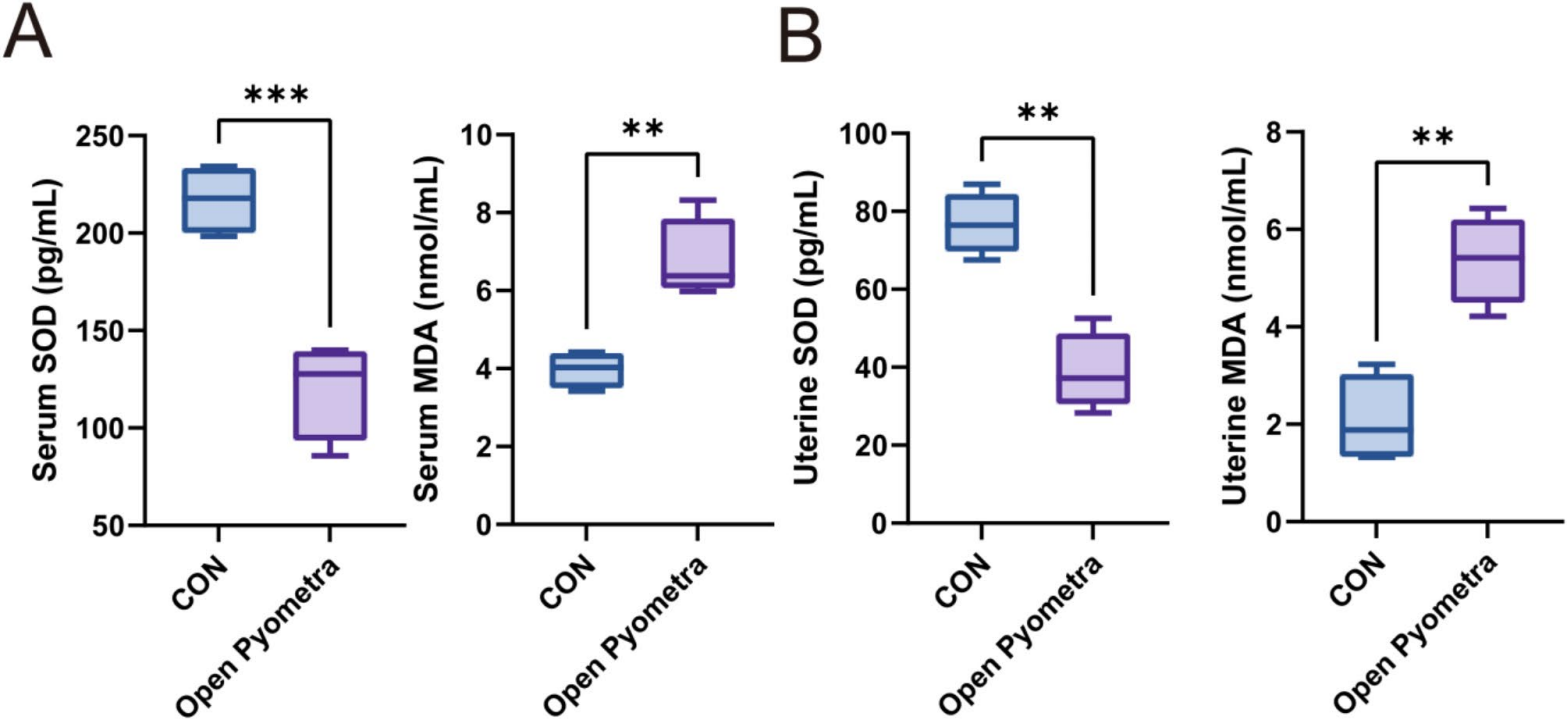
Evaluation of SOD and MDA activities in serum and tissues samples of healthy bitches and dogs with pyometra ( ** *p* < 0.01; *** *p* < 0.001)

Besides, in comparison to healthy bitches, pyometra group samples exhibited increased MDA level and decreased SOD activity in serum and uterine tissue (Fig. 1 and supplementary data).

#### Uterine tissue histopathology and immunohistochemistry analysis

**Fig. 2:**
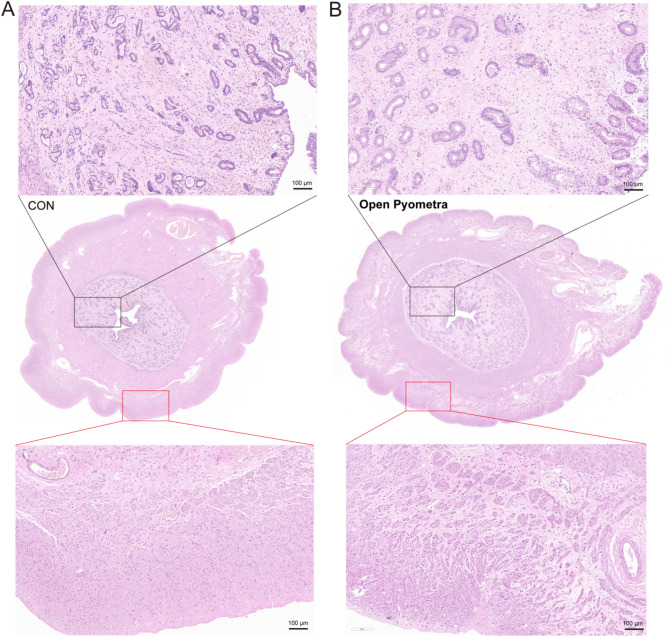
HE histopathology. (**A**) Uterine horn histopathology in healthy bitches (**B**) histopathology image of uterine tissue sample in canines pyometra. The scale bar of the magnified viewing angle is 100 μm

**Fig. 3:**
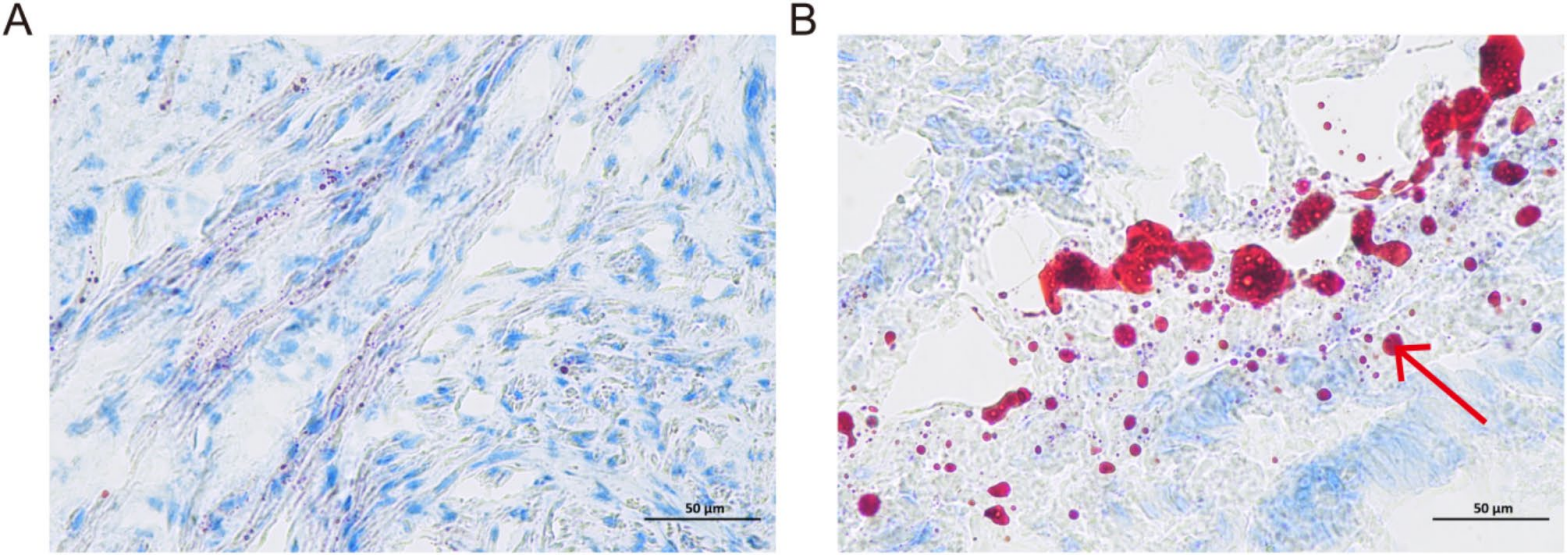
ORO staining showed accumulated lipid droplets of endometrium in healthy bitches (**A**) and in dogs with pyometra (**B**) (red arrow)

When uterine histopathology was examined by HE staining (Fig. 2), significant interstitial edema and gland hyperplasia were observed in dogs with pyometra. Besides, the presence of accumulated lipid was observed in the uterine tissue of bitches with pyometra (Fig. 3). Oil Red O staining analysis of uterine tissue shows varying staining intensities shown in Table 1.

**Table 1:** Oil red O staining analysis of uterine tissue samples in healthy dogs and dogs with pyometra

Oil Red O	No staining (0) % healthy/pyometra	Low (1) % healthy/pyometra	Moderate (2) % healthy/pyometra	Strong (3) % healthy/pyometra
Uterine tissue	89.20/54.35	8.82/7.59	1.46/6.58	0.52/31.48

**Fig. 4:**
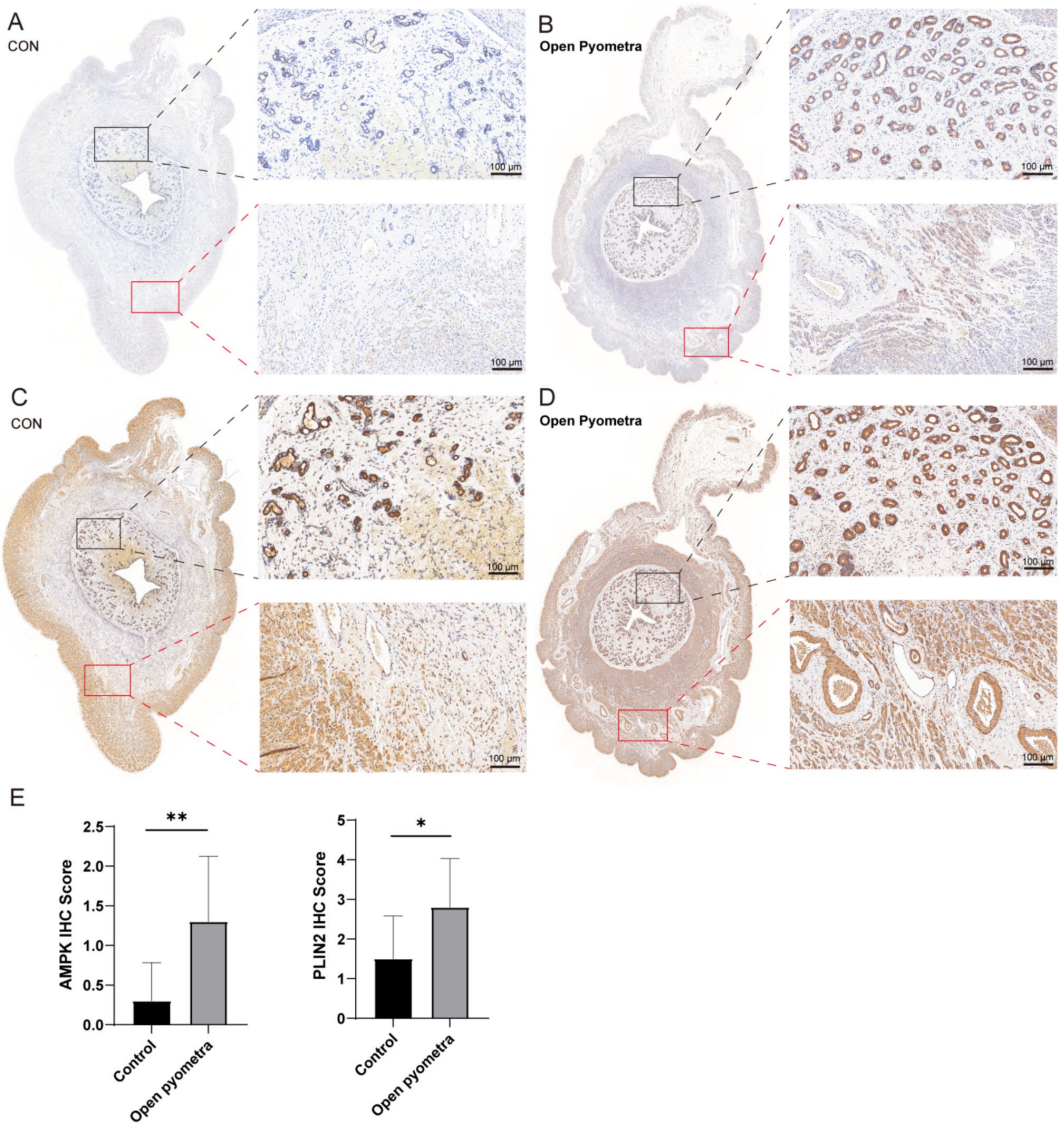
Immunohistochemical analysis of AMPK (**A**, **B**) and PLIN2 (**C**, **D**) in paraffin sections. The expression of p-AMPK and PLIN2 in healthy controls and pyometra affected uterine tissue samples was examined using immunohistochemistry (IHC) at x20 (100 μm) magnification with Aperio ScanScopev12.4.6 digital scanning analysis of slides; (**E**) IHC staining score (* *p* < 0.05; ** *p* < 0.01)

Immunohistochemical analysis showed a significant increase in the expression of p-AMPK and PLIN2 in the myometrium, surface epithelium, and glandular epithelium of pyometra group samples compared to the control group (Fig. 4).

#### Western blot analysis

**Fig. 5:**
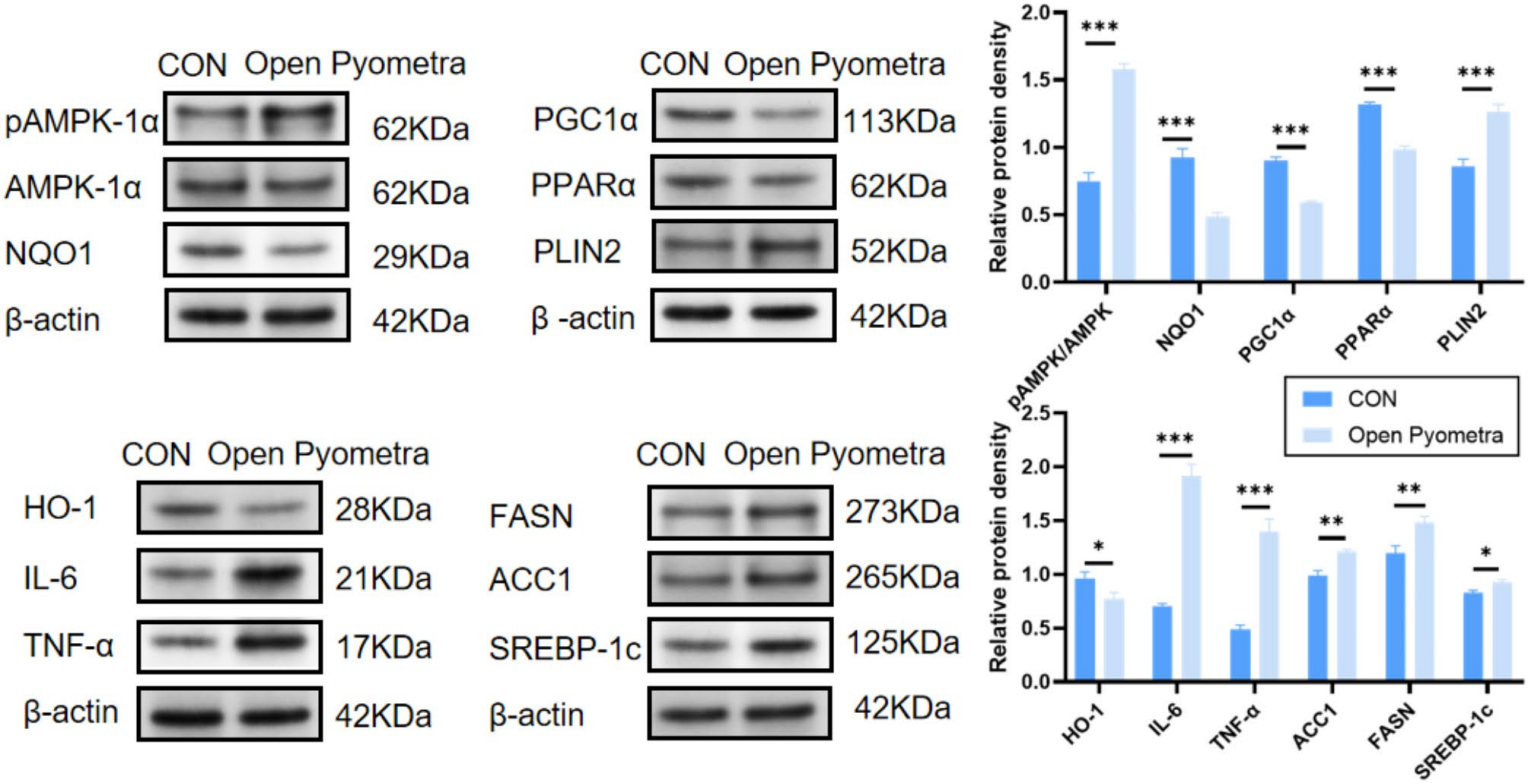
Western blot detection of factors related to inflammation, oxidative stress, and lipid metabolism in healthy and pyometra affected canine uterine tissues (* *p* < 0.05; ** *p* < 0.01; *** *p* < 0.001)

In the investigation of uterine samples of pyometra group, we observed significant alterations in the expression of a series of genes associated with metabolism and inflammation, unveiling intricate biological responses in uterine tissue under pathological conditions. These alterations encompassed an upsurge in the energy metabolism regulator pAMPK-α, implying that cells exhibit an adaptive response to metabolic stress. The upregulation of immune markers IL-6 and TNF-α demonstrated a robust immune reaction. Concurrently, reductions in NQO1 and HO-1 indicated impaired antioxidant function, rendering tissues more susceptible to oxidative damage. Regarding metabolism, the elevation of lipid synthesis-related proteins ACC1, FASN, SREBP-1c and PLIN2 signified heightened lipid synthesis activity. This could be attributed to increased cellular demand for energy and structural molecules amidst inflammation and metabolic stress. Conversely, diminished levels of PPARα and PGC1α suggested a declining trend in lipid oxidation and energy metabolism which may lead to decreased fatty acid oxidation function further exacerbating metabolic imbalance (Fig. 5).

### Experiment 2

#### Establishment of a canine endometrial epithelial cell model for determining lipid accumulation and inflammation

**Fig. 6:**
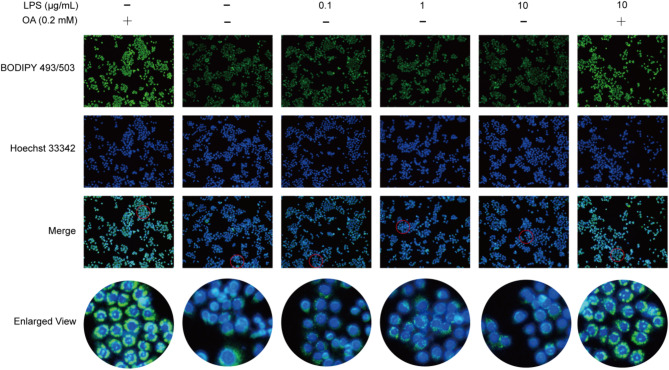
Illustrates the alterations in immunofluorescence of lipid droplets in canine endometrial epithelial cells following treatment with different concentrations of LPS and OA. (Scale bars = 200 μm)

To simulate uterine inflammation and assess lipid droplet status, a model was established using LPS + OA. Treatment with different concentrations of LPS did not lead to an increase in LDs; however, cells supplemented with oleic acid exhibited significantly enhanced lipid drop-positive signaling. Therefore, we opted for the utilization of LPS + OA to construct a model that accurately represents lipid droplet accumulation and inflammation. In addition, by local magnification maps, it was observed that inconsistent distribution of lipid droplets in the periphery of the nucleus in the LPS-stimulated versus control group (Fig. 6).

#### The impact of AMPK and PLIN2 on inflammation, oxidative stress, and lipid metabolism in canine endometrial epithelial cells

**Fig. 7:**
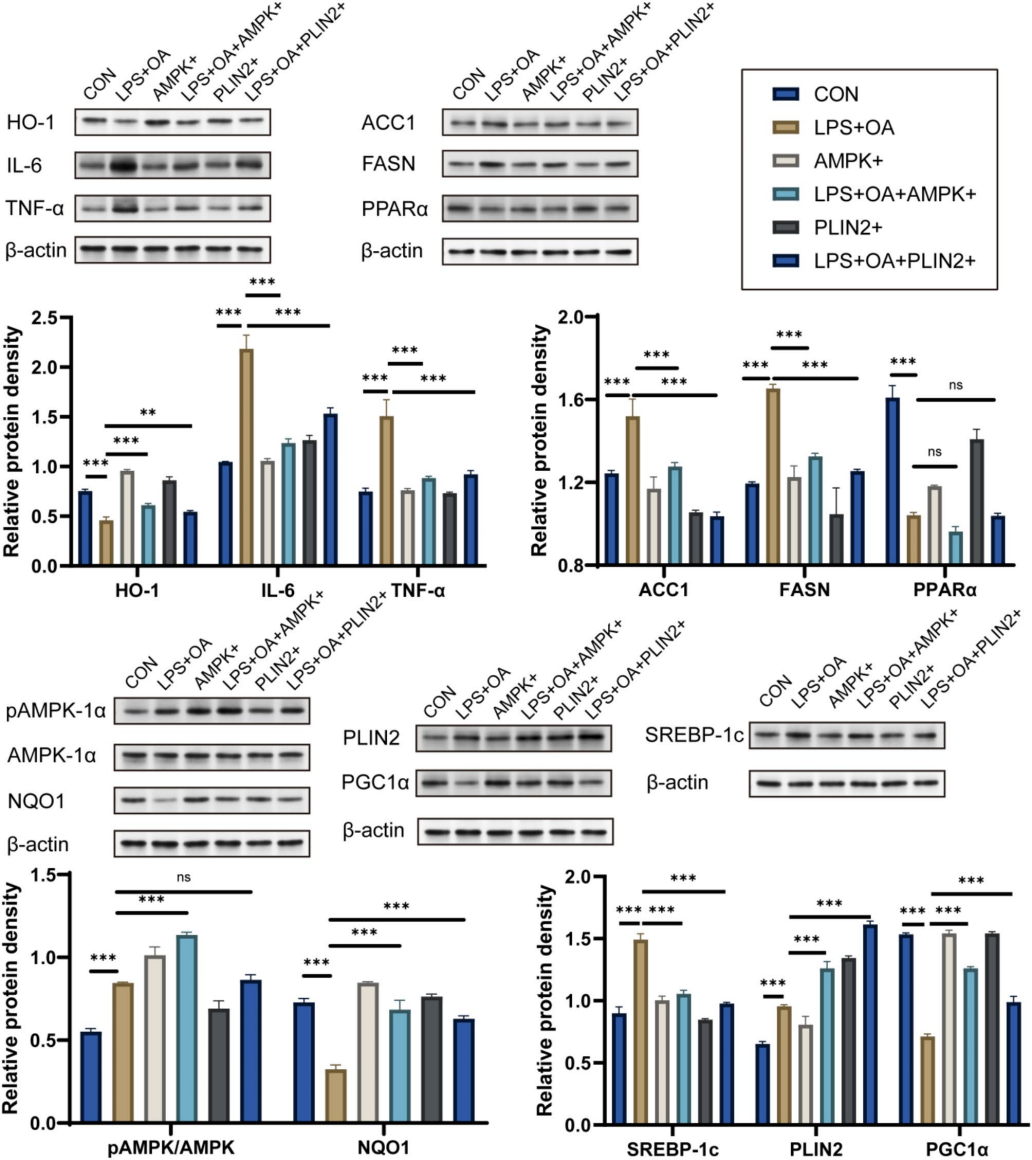
The expression of inflammation, oxidative stress, and lipid metabolism-related factors in canine endometrial epithelial cells was assessed using Western blot analysis (ns = no significant; ** *p* < 0.01; *** *p* < 0.001)

To investigate the regulatory role of AMPK and PLIN2 in inflammation and oxidative stress induced by LPS and OA in endometrial epithelial cells, we designed the following experimental groups: Control group (CON), LPS + OA treatment group (LPS + OA), PLIN2 overexpression group (PLIN2+), LPS + OA combined with PLIN2 overexpression group (LPS + OA + PLIN2+), AMPK activation group (AMPK+), and LPS + OA combined with AMPK activation group (LPS + OA + AMPK+). The results demonstrated a significant increase in the expression levels of inflammatory markers IL-6 and TNF-α under LPS and OA treatment, accompanied by a notable decrease in NQO1 and HO-1 expressions. However, both AMPK and PLIN2 overexpression were found to alleviate this condition (Fig. 7).

## Discussion

In this study, we investigated the pivotal role of lipid metabolism in the pathophysiological process of canine pyometra. Previous studies clearly showed that lipid metabolism not only serves as an energy source for the body but also plays a crucial role in early pregnancy establishment and maintenance, as well as being involved in the regulation of various diseases [25–27]. Particularly within the context of uterine physiology and pathology, increasing attention has been directed towards lipid alterations [26, 28, 29]. Through non-targeted metabolomics analysis, Zheng et al. [30] have discovered that lipid metabolism represents one of the major differential metabolic pathways in the endometrium, suggesting its potential significance in the pathological progression of pyometra. Although there are certain limitations in the present study, such as a restricted sample size, our results have demonstrated that the significant increase in lipid droplets within uterine tissue is consistent with previous studies [10]. However, specific regulatory mechanisms implicated in the advancement of pyometra and their involvement in relevant changes remain incompletely understood.

Superoxide dismutase (SOD) is one of the most important antioxidant enzymes [31]. Increased inflammation has been associated with a reduction in SOD activity [32]. The present study demonstrates that dogs suffering from pyometra exhibit decreased SOD activity in both uterine tissue and serum, as previously reported [6]. Additionally, elevated levels of MDA in blood and uterine tissue have been documented, suggesting that MDA could serve as a potential biomarker for assessing the severity of pyometra. These findings highlight the interplay between oxidative stress and inflammation in the pathophysiology of pyometra.

The canine pyometra is accompanied by immune activation and severe inflammatory infiltration [33]. As a major organelle involved in inflammatory responses, LDs accumulate in microglia, leading to age-related brain dysfunction and promoting inflammation [34]. Macrophages can significantly increase LDs accumulation and triglyceride (TG) levels under LPS/IFN-γ stimulation [35]. However, we observed that LPS stimulation had no effect on LDs formation and quantity in canine endometrial epithelial cells medium, which may be attributed to differences in inflammatory response and metabolic requirements among different cell types. Overexpression of LDs is involved in regulating genes related to lipid metabolism synthesis and decomposition during the physiological and pathological processes of the uterus [10]. Therefore, this study further investigated whether inflammatory stimulation affects the expression level of lipid-related proteins in endometrial epithelial cells when LDs accumulation occurs. Western blot analysis revealed significant changes in lipid synthesis and protein breakdown in canine pyometra samples. Specifically, ACC1, FASN, SREBP-1c, and PLIN2 expression increased while PPARα and PGC1α expression decreased. These alterations in lipid metabolism may contribute to cell survival and function under an inflammatory context while also influencing regulation of the inflammatory response through lipid-mediated signaling pathways. Importantly, peroxisome proliferator-activated receptors (PPARs), belonging to the nuclear receptor family, play a crucial regulatory role in uterine development and function. Among them, PPARα plays a key role in regulating lipid metabolism with anti-inflammatory properties [36, 37]. PGC1α is typically downregulated by various inflammatory mediators and cytokines; this downregulation is closely associated with the uterine inflammatory state [38, 39]. The uterine tissue samples of dogs with pyometra exhibits severe inflammatory infiltration, which can potentially result in the downregulation of PPARα and its co-activator PGC-1α. Given the crucial roles that PPARα and PGC1α play in oxidative stress, lipid metabolism, and inflammation [39, 40], gaining a deeper understanding of the specific regulatory mechanisms governing PPARα and PGC-1α in pyometra will be pivotal for unraveling the nature of uterine pathology.

AMPK regulates the expression of PPARα and PGC1α, thereby mitigating mitochondrial dysfunction and oxidative stress while maintaining metabolic homeostasis [41, 42]. As a pivotal cellular energy-sensing enzyme, AMPK is involved in lipid metabolism regulation and essential for normal female reproductive function [17, 43]. Generally, activation of AMPK is associated with enhancing cellular energy status and preserving metabolic balance. Reduced AMPK expression may lead to increased markers of cytotoxicity, inflammation, and oxidative stress. Conversely, upregulation of AMPK can inhibit these detrimental reactions [44]. Immunohistochemistry and Western blot analysis revealed a significant increase in AMPK expression in spontaneously developed canine pyometra.

To further investigate the effects of AMPK and PLIN2 on inflammation and oxidative stress, as well as their impact on lipid accumulation and inflammatory infiltration, we stimulated canine endometrial epithelial cells with LPS + OA to model the cellular inflammatory response and excessive fat accumulation [45]. In addition to assessing protein expression related to LDs formation and genes associated with lipid metabolism, we examined whether the use of an AMPK activator and PLIN2 overexpression could mitigate the changes in inflammation, oxidative stress and lipid metabolism induced by LPS + OA treatment. Increased PLIN2 expression reduces oxidative stress and alters lipid metabolism in fibroblasts and gastric cancer cells [12, 46, 47]. Our findings demonstrate that activating AMPK and enhancing PLIN2 can effectively suppress the expression of inflammatory factors such as TNF-α and IL-6, restore antioxidant enzyme levels (NQO1 and HO-1), as well as regulate the expression of factors like PGC1α that govern lipid metabolism. This suggested that through modulation of lipid metabolism and oxidative stress, AMPK and PLIN2 may alleviate inflammatory responses occurring in the endometrium. While our study highlights the significant role played by AMPK and PLIN2 in regulating interactions between inflammatory responses, immune disorders in the endometrium, as well as localized infection-induced changes at a small scale within this environment; However, further exploration is required to fully comprehend their specific mechanisms/functions within this field.

## Conclusion

This study demonstrates that dysregulated lipid metabolism is a critical driver of canine pyometra progression, characterized by coordinated upregulation of ACC1, FASN, SREBP-1c, PLIN2 and suppression of PPARα and PGC1α. Mechanistically, AMPK/PLIN2 activation emerges as a key regulatory node, attenuating inflammatory responses through TNF-α and IL-6 suppression and restoring redox homeostasis via NQO1 and HO-1 induction. While these findings nominate AMPK and PLIN2 as potential therapeutic targets, validation in expanded, multicenter cohorts is warranted to confirm clinical translatability.

## Electronic supplementary material

Below is the link to the electronic supplementary material.

Supplementary Material 1

Supplementary Material 2

## Data Availability

Data is provided within the manuscript or supplementary information files.
