# Supplementary figures and images for: Expression of AMPK and PLIN2 in the regulation of lipid metabolism and oxidative stress in bitches with open cervix pyometra
Source: BMC Vet Res. 2025 Mar 13;21:164. doi: 10.1186/s12917-025-04622-1 (PMC11905669; doi:10.1186/s12917-025-04622-1)

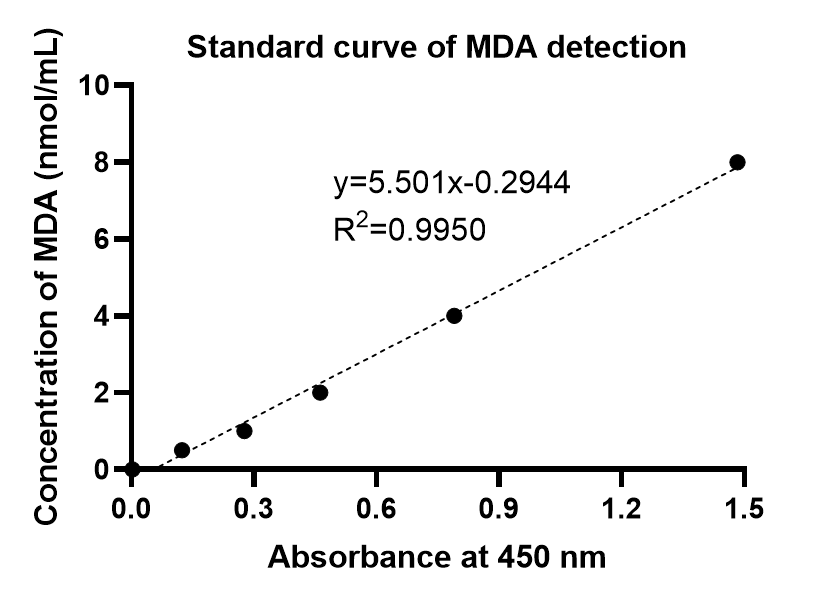


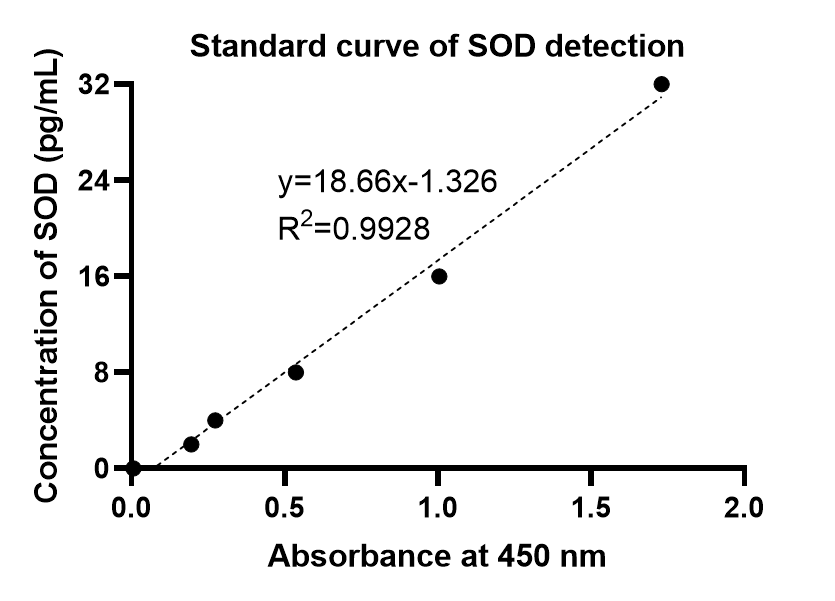

Supplement: Supplementary file 1 — Supplementary Material 1 [file 12917_2025_4622_MOESM1_ESM.docx]
