# Supplementary material for: Expression of AMPK and PLIN2 in the regulation of lipid metabolism and oxidative stress in bitches with open cervix pyometra
Source: BMC Vet Res. 2025 Mar 13;21:164. doi: 10.1186/s12917-025-04622-1 (PMC11905669; doi:10.1186/s12917-025-04622-1)
Supplement: Supplementary file 2 — Supplementary Material 2 [file 12917_2025_4622_MOESM2_ESM.pdf]

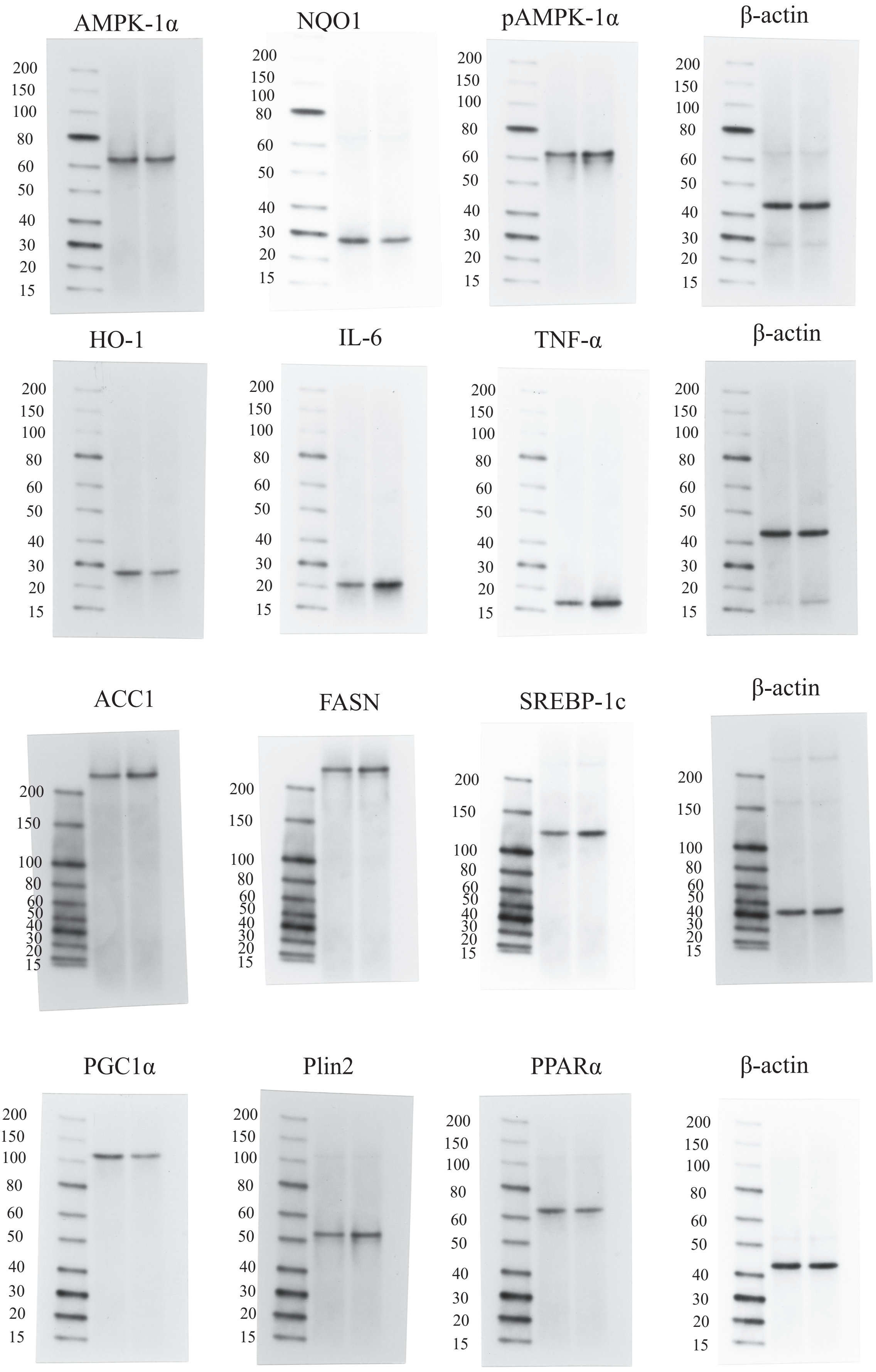
AMPK-1α
200 150 100 80 60 50 40 30 20 15
NQO1
200 150 100 80 60 50 40 30 20 15
pAMPK-1α
200 150 100 80 60 50 40 30 20 15
β-actin
200 150 100 80 60 50 40 30 20 15
HO-1
200 150 100 80 60 50 40 30 20 15
IL-6
200 150 100 80 60 50 40 30 20 15
TNF-α
200 150 100 80 60 50 40 30 20 15
β-actin
200 150 100 80 60 50 40 30 20 15
ACC1
200 150 100 80 60 50 40 30 20 15
FASN
200 150 100 80 60 50 40 30 20 15
SREBP-1c
200 150 100 80 60 50 40 30 20 15
β-actin
200 150 100 80 60 50 40 30 20 15
PGC1α
200 150 100 80 60 50 40 30 20 15
Plin2
200 150 100 80 60 50 40 30 20 15
PPARα
200 150 100 80 60 50 40 30 20 15
β-actin
200 150 100 80 60 50 40 30 20 15

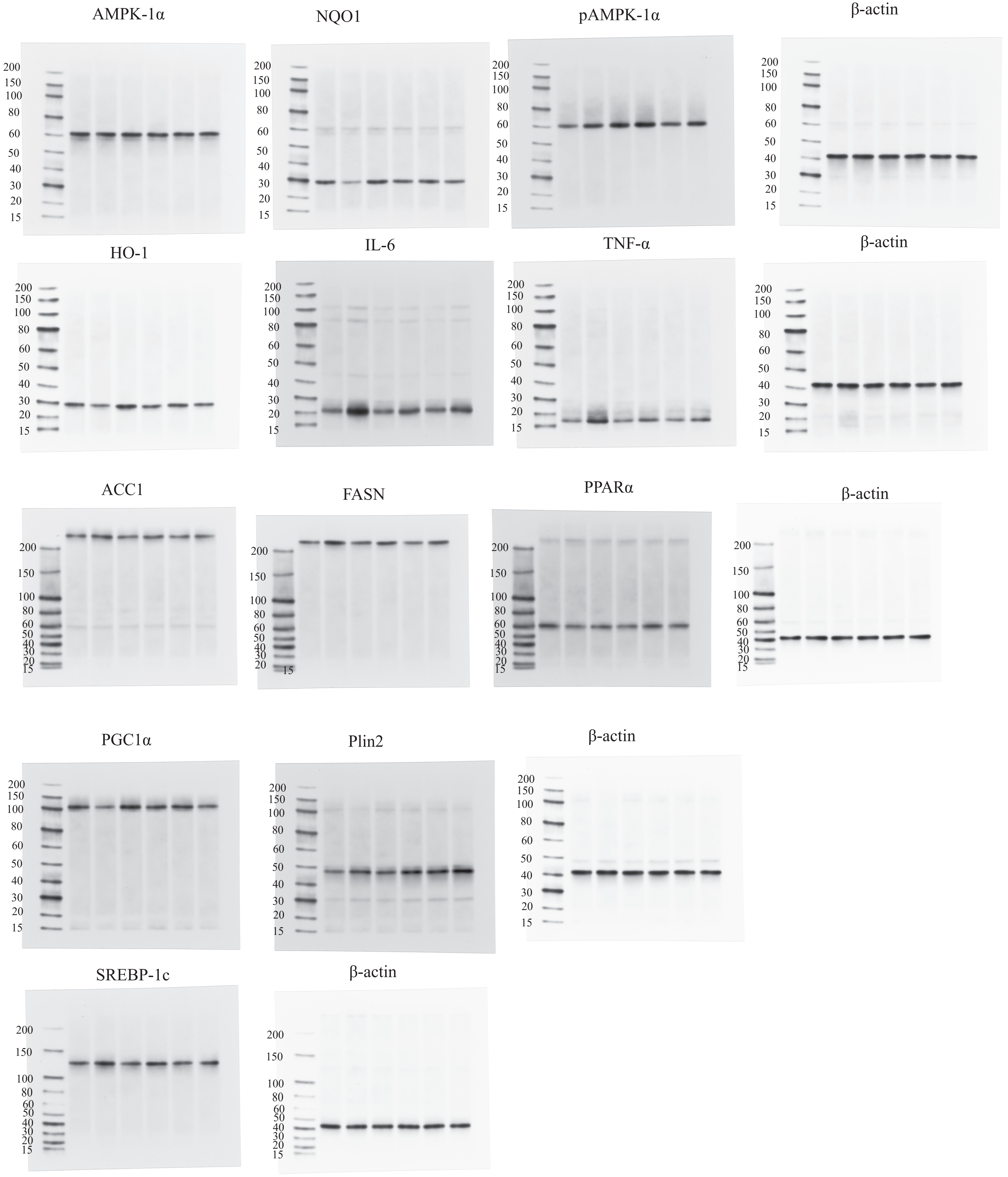
AMPK-1α
NQO1
pAMPK-1α
β-actin
HO-1
IL-6
TNF-α
β-actin
ACC1
FASN
PPARα
β-actin
PGC1α
Plin2
β-actin
SREBP-1c
β-actin
